# Low Dose of Direct Oral Anticoagulants after Left Atrial Appendage Occlusion

**DOI:** 10.3390/jcdd8110142

**Published:** 2021-10-28

**Authors:** Pedro Luis Cepas-Guillen, Eduardo Flores-Umanzor, Ander Regueiro, Salvatore Brugaletta, Cristina Ibañez, Laura Sanchis, Marta Sitges, Josep Rodés-Cabau, Manel Sabaté, Xavier Freixa

**Affiliations:** 1 Cardiology Department, Cardiovascular Institute (ICCV), Hospital Clinic, IDIBAPS, University of Barcelona, 08036 Barcelona, Spain; cepas@clinic.cat (P.L.C.-G.); ejfu0209@gmail.com (E.F.-U.); AREGUEIR@clinic.cat (A.R.); SABRUGAL@clinic.cat (S.B.); LSANCHIS@clinic.cat (L.S.); MSITGES@clinic.cat (M.S.); josep.rodes@criucpq.ulaval.ca (J.R.-C.); MASABATE@clinic.cat (M.S.); 2 Anesthesiology Department, Hospital Clinic, IDIBAPS, University of Barcelona, 08036 Barcelona, Spain; CRIBANEZ@clinic.cat; 3 Quebec Heart and Lung Institute, Quebec City, QC G1V 4G5, Canada

**Keywords:** atrial fibrillation, non-vitamin K antagonist oral anticoagulants, left atrial appendage occlusion, bleeding

## Abstract

The optimal antithrombotic strategy following left atrial appendage occlusion (LAAO) is not yet clearly established. Low-dose non-vitamin K antagonist oral anticoagulants (NOAC) might represent a valid alternative, but data regarding their usage is scarce. The aim of this study was to examine the efficacy and safety of low-dose NOAC compared to single (SAPT) or dual antiplatelet therapies (DAPT) after LAAO. We included consecutive patients with non-valvular atrial fibrillation who underwent LAAO and received low-dose apixaban, SAPT, or DAPT at discharge. The primary objective of this study included an efficacy endpoint (thromboembolic events and device related thrombosis (DRT)) and a safety endpoint (incidence of major bleeding) within the first three months after LAAO. A total of 139 patients were included. This group involved SAPT in 26 (18%), DAPT in 73 (53%), and apixaban in 40 (29%) patients. Follow-up at three-months showed no significant differences in the primary efficacy endpoint (2 (8%) SAPT, 3 (4%) DAPT and 0 (0%) apixaban; *p* value = 0.25). In contrast, the primary safety endpoint occurred more frequently in DAPT patients (7 (10%) DAPT, 0 (0%), SAPT and 0 with apixaban; *p* value = 0.03). Combining both efficacy and safety outcomes, low dose apixaban had a lower rate of events (2 (8%) with SAPT, 9 (12%) with DAPT and 0 (0%) with apixaban; *p* = 0.046). Low-dose apixaban after LAAO may be a valid alternative to DAPT and SAPT as depicted by the reduction in the occurrence of major bleedings and combined DRT/major bleedings respectively. Randomized data will be necessary to validate this strategy.

## 1. Introduction

It has been estimated that 6–12 million people worldwide will suffer non valvular atrial fibrillation (NVAF) in the US by 2050, and 17.9 million people will experience this condition in Europe by 2060 [1]. Atrial fibrillation is a major risk factor for ischemic stroke and represents an important economic burden along with significant morbidity and mortality [2]. Oral anticoagulation (OAC) therapy is the standard of care to prevent thrombus formation and cardioembolic events in patients with NVAF [3]. However, about 10 to 20% of patients with NVAF have an absolute or relative contraindication to oral anticoagulation therapy due to increased risk of bleeding [4]. Percutaneous left atrial appendage occlusion (LAAO) has demonstrated to be an alternative for the prevention of cardioembolic events in these patients [5,6] while avoiding long-term risks of OAC. Nevertheless, LAAO devices require short-term (one to three months) postprocedural oral antithrombotic therapy to prevent device-related thrombosis (DRT). Currently, there is no consensus regarding the optimal antithrombotic treatment strategy for DRT prevention, dual antiplatelet therapy (DAPT) being the most widely used strategy. In addition, the high hemorrhagic risk in most of patients makes this decision even more challenging [7]. Among non-vitamin K antagonist oral anticoagulants (NOAC), apixaban has shown a remarkable safety and efficacy profile with low rates of ischemic and hemorrhagic events compared to vitamin K antagonist (VKA) or single antiplatelet therapy (SAPT) [8]. However, preliminary data regarding its use after LAAO is scarce. Therefore, the aim of our study was to examine the efficacy and safety of low-dose NOAC compared to single antiplatelet therapy (SAPT) or DAPT after LAAO.

## 2. Materials and Methods

### 2.1. Patient Selection and Follow-Up

The study included consecutive patients with NVAF who underwent LAAO in our institution between 2012 and September 2020. For the purpose of the study, patients were divided in three groups according to the antithrombotic treatment at discharge: SAPT (Aspirin 100 mg o.d), DAPT (Aspirin 100 mg o.d + Clopidogrel 75 mg o.d), or low-dose NOAC (apixaban 2.5 mg b.i.d). The use of low-dose apixaban was independent of meeting dose reduction criteria (two criteria from: age ≥80 years, body weight ≤60 kg, or serum creatinine ≥1.5 mg/dL (133 umol/L)). Thus, no patients on full-dose apixaban were included. The choice of treatment was based on the criteria of the treating physician. Patients without antithrombotic treatment or with low-molecular-weight heparin or VKA at discharge were excluded. Anticoagulant treatment was withdrawn the day before the procedure. During the procedure, a weight-adjusted bolus of unfractionated heparin (70–100 IU/kg) was administered immediately after crossing the interatrial septum. The follow-up protocol included a clinical control at three months after hospital discharge. The first imaging follow-up with transesophageal echocardiography (TEE), was performed between the sixth and twelfth week. Only in patients with contraindication or intolerance to the TEE probe, cardiac tomography (CT) was performed. A satisfactory result on TEE (complete LAAO in the absence of DRT) allows withdrawing one antiplatelet agent, in case of patients on DAPT, or the low-dose apixaban, modifying the antithrombotic therapy unless otherwise indicated. In this sense, SAPT with aspirin is the most frequent antithrombotic treatment after the first three-months after LAAO and is usually continued indefinitely. The study was approved by the Ethics Committee of our center and consistent to the principles outlined in the Declaration of Helsinki. Prospectively collected data were transferred to a dedicated anonymized database. All patients signed informed written consent before the procedure.

### 2.2. Study Endpoints and Definitions

Details regarding LAAO procedure and special features of the occlusion devices have been published elsewhere [9]. LAAO was performed under TEE, fluoroscopic guidance, and general anesthesia. Femoral venous access was obtained in the right femoral vein in our patients, as it allowed an easier and more precise transseptal puncture, and in order to avoid vascular access, usually located in upper extremities. Transseptal puncture was performed under fluoroscopic and TEE guidance in the inferior and posterior part of the fossa ovalis using a BRK-0 (minor curve) or BRK-1 (large curve) needle. Once the trans septal sheath was advanced in the left atrium, pericardial effusion was ruled out by echocardiographic evaluation. Through the transseptal sheath, a 5 Fr pigtail marker catheter was then advanced into the LAA to perform accurate angiographic left atrial appendage measurements in “real-time” to evaluate the size of the device. Then, a 10 or 14 Fr delivery catheter was inserted, and the device was deployed. After deployment of the occlusion device and before complete release, the device stability was ensured. The stability test with gentle backwards tension was done. Subcutaneous suture with a “figure of 8” was the preferred method to achieve proper hemostasis in our case series. Procedural success was defined as successful implantation of the device in the LAA [10]. Procedural adverse events, major adverse events (MAEs), and DRT were reported according to the Munich Consensus paper [10]. Major bleeding events were defined as type 3 or greater on the Bleeding Academic Research Consortium (BARC) scale [11]. Adverse events reported at follow-up included death (cardiovascular and non-cardiovascular), stroke, systemic embolism, any bleeding (major and minor) and DRT. The primary objective included an efficacy endpoint (thromboembolic events and DRT) and safety endpoint (of major bleeding) during the first three months after LAAO. Secondary endpoints included the individual components of the primary endpoint, any bleeding (major and minor), and mortality (cardiovascular and non-cardiovascular).

### 2.3. Statistical Analysis

Categorical variables are presented as frequencies (percentages), assessing the differences by Chi-square test (or Fisher test when necessary). Continuous variables are presented as a mean ± standard deviation (SD) or as a median (interquartile range). The Kolmogorov-Smirnov test was applied to ensure normal distribution. Continuous variables were compared using Student’s *t*-test or the Mann-Whitney U test, as appropriate. For all analyses, a two tailed *p*-value < 0.05 was used as the criterion for statistical significance. Follow-up was considered to terminate at three-months follow-up. Analyses were performed using STATA software (V 14.0, StataCorp LP, College Station, TX, USA).

## 3. Results

**Figure 1:**
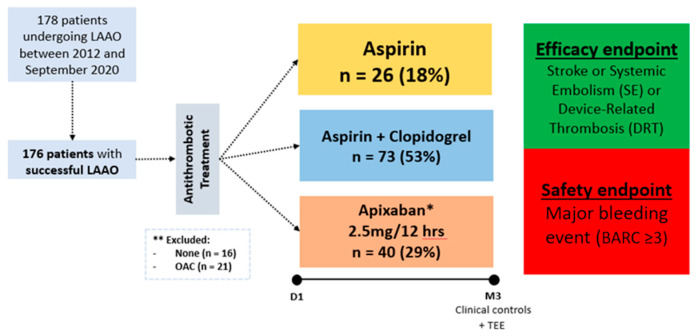
Study Flowchart. January 2012 and September 2020, 139 patients with successful LAAO implantation were included. LAAO, left atrial appendage occlusion; OAC, oral anticoagulation.

Between January 2012 and September 2020, 178 patients underwent LAAO in our institution. The flowchart of the study is presented in Figure 1. In 176 (98.8%) patients, LAAO was successfully performed. Patients with SAPT, DAPT, or low-dose NOAC at discharge after the procedure were included. Thirty-seven patients were excluded due to OAC (*n* = 21) or no antithrombotic treatment (*n* = 16) after LAAO. Thus, a total of 139 patients were included in the analysis: SAPT was administered in 26 (18%), DAPT in 73 (53%), and apixaban in 40 (29%) of patients. Baseline characteristics are shown in Table 1. No significant differences in baseline characteristics were observed other than a higher prevalence of previous stroke in the low-dose NOAC and SAPT groups compared to DAPT. Of note, the baseline hemorrhagic risk was high (mean HAS-BLED score 3.6 ± 1.0), with no difference among groups.

As shown in Table 2, the most common LAAO occluder was the Amplatzer Amulet/Cardiac Plug (81%), followed by Lambre (17%), and Watchman devices (2%). No differences in procedural outcomes were observed among device groups. The rate of procedural MAEs was 4% (major bleeding and/or vascular access complications) and no device embolization, cardiac tamponade or mortality was reported.

**Figure 2:**
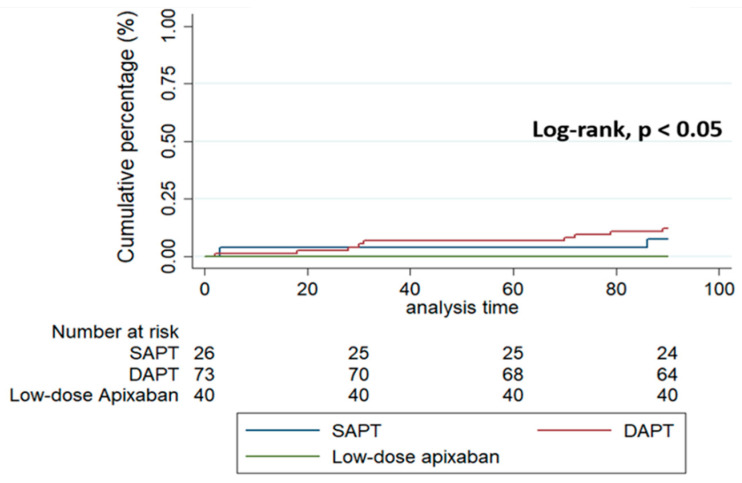
Kaplan-Meier curve of composite endpoint (efficacy and safety endpoints).

Clinical outcomes at three-month follow-up are shown in Table 3 and Figure 2. The primary efficacy endpoint (stroke, systemic embolization, or DRT) occurred in wo (8%) patients with SAPT, three (4%) with DAPT and zero (0%) with low-dose NOAC (*p* value = 0.25). The primary safety endpoint (major bleeding) occurred exclusively in seven (10%) patients in the DAPT group (*p* value = 0.03). The composite endpoint (efficacy + safety endpoints) occurred in 11 patients (8%): 2 patients (8%) with SAPT, 9 patients (12%) with DAPT and 0 (0%) with low-dose NOAC (*p* value = 0.046). Regarding secondary endpoints, no differences between groups were observed other than a trend towards a higher incidence of any bleeding (major and minor) in the DAPT group. No differences were detected in terms of the development of DRT and the device of type (two DRT with ACP device, two with Amulet device and one with Lambre device). This trend towards a higher incidence of DRT or major bleeding in DAPT was observed at 12-months follow-up compared to low-dose apixaban (Table S1, Figure S1).

## 4. Discussion

The main findings of the present study were that after the assessment of antithrombotic strategies after LAAO: (1) low-dose NOAC was associated with a lower incidence of major bleedings compared to DAPT and SAPT; and (2) low-dose NOAC showed a similar efficacy profile compared to SAPT and DAPT in terms of thromboembolism prevention and DRT occurrence.

### 4.1. Low-Dose NOAC Showed a Better Safety Profile Compared to SAPT or DAPT

Despite the growing evidence in support of LAAO, several questions remain unanswered in this field. One of the most relevant is the optimal antithrombotic strategy following a successful procedure. The ideal antithrombotic therapy after LAAO should provide a balance between efficacy (for the prevention of embolic events and DRT) and safety (for the occurrence of major bleeding) as patients undergoing LAAO generally show a high risk of thromboembolic (but also hemorrhagic events). In our study, low-dose NOAC with apixaban showed a better safety profile, as depicted by the lower rate of major bleedings as compared to SAPT or DAPT. Currently, LAAO is mainly indicated in NVAF patients with previous hemorrhagic events and OAC or patients deemed at high risk for bleeding. Indeed, major bleeding events following LAAO are not uncommon in this population and often range between 5 and 10% within the first year [12] and being associated with increased mortality [13]. In this sense, efforts should be directed towards reducing the incidence of bleedings at follow-up by modulating antithrombotic therapy at discharge. The optimal antithrombotic therapy after LAAO is not well-established due to the lack of supporting randomized evidence and significant heterogeneity for the individual risk of bleeding of every patient. In this sense, the current consensus statement on optimal post-interventional antithrombotic drug regimen after LAAO recommends that the antithrombotic regimen should be tailored individually due to the majority of patients subjected to LAAO are at high risk for bleeding and the lack of evidence [14]. Although the main randomized clinical trials [15,16] included warfarin in their antithrombotic therapy (warfarin and aspirin (81 mg/d) for 45 days, followed by aspirin (325 mg/d) and clopidogrel (75 mg/d) for 6 months, and then aspirin (325 mg/d) alone), data from real observational studies indicated that DAPT for three months is the most used antithrombotic therapy after LAAO [17]. The usage of DAPT after LAAO is based on prior experience with coronary stents, foramen ovale, and atrial septal occlusion devices. However, despite being associated with a low rate of stroke and DRT, as the ASAP study demonstrated [18], DAPT does not prevent the risk of major bleeding events following LAAO [14,16,17]. Both PROTECT-AF and PREVAIL were designed in the era before the availability of NOAC agents, and the introduction of these drugs has opened up a new spectrum of therapeutic options. Recently, Faroux et al. performed a multicenter analysis that included 592 consecutive patients with a relative contraindication to OAC who underwent LAAC and received either DAPT or DOAC for 1–3 months [19]. Each patient receiving DOAC was matched with two patients on DAPT based on propensity-score (propensity-matched population of 285 patients). They found that, within the first three months following LAAO, a numerically higher rate of non-procedural related bleedings (7.4% vs. 3.2%) were observed in DAPT patients. Our results are concordant, showing a higher incidence of major bleedings in the DAPT group. Interestingly, the absence of major bleedings in our NOAC group may respond to the fact that we use low dose instead of full dose NOAC. This benefit may extend to different high-risk populations, usually under-represented in major clinical trials, such as patients with chronic kidney disease. In the latter situation, although chronic kidney disease is associated with a prothrombotic state and a high risk of bleeding [20], LAAO could be a valid alternative to OAC [21].

### 4.2. Low-Dose NOAC Showed a Similar Efficacy Profile Compared to SAPT or DAPT

In addition to the safety profile, low-dose apixaban could also represent a valid alternative to DAPT in terms of efficacy in preventing thromboembolic events and DRT until LAAO endothelization occurs. Device related thrombosis is currently one of the most concerning complications in the LAAO field which occurs in, at least, 4% of patients following LAAO and is associated with a four- to five-fold increase in ischemic events [22]. Nonetheless, the true incidence of DRT might be difficult to assess due to the variations in surveillance protocols. DRT is caused by the formation of thrombus over the atrial aspect of the occluding device before its complete endothelization, which typically takes about 90 days [7]. One possible explanation for DRT may respond to the increase in prothrombin fragment 1 + 2 and thrombin-antithrombin III following the procedure, with no increase in platelet activation [23]. Another hypothesis concerning DRT may imply a low flow situation such as observed in large veins or atrial/cardiac chambers. Both premises might suggest that apixaban could be more appropriate than any antiplatelet therapy to reduce this thrombotic risk. In fact, our hypothesis that low-dose apixaban is a valid strategy to prevent DRT while keeping a low risk of bleeding is based on two facts: (1) excluding the LAA from the circulation would eliminate the main reservoir of thrombus from the body, so lower anticoagulation dose should be indeed necessary; and (2) the successful treatment of DRT with low-dose apixaban observed by our group in previous reports [24].

Although our preliminary data about the use of low-dose NOAC in this challenging setting are favorable, they should be considered as hypothesis-generating, especially given that NOACs failed to demonstrate a higher efficacy and safety in patients with mechanical heart valves, associating with an increased rate of thromboembolic and bleeding complications, as compared with VKA [25] and some cases showed an increase risk of DRT with NOAC in percutaneous patent foramen ovale closure [26]. Thus, this post-LAAO antithrombotic approach needs to be validated in randomized clinical trials. The ongoing ADALA (randomized clinical trial to compare two antithrombotic strategies after left atrial appendage occlusion: double antiplatelet therapy vs. apixaban) study (EudraCT number: 2018-001013-32) [27] and ANDES (‘Short-Term Anticoagulation Versus Antiplatelet Therapy for Preventing Device Thrombosis Following Left Atrial Appendage Closure’) trial (NCT03568890) should confirm this hypothesis.

### 4.3. Limitations

The main limitation of this study is its observational design, which implies an inherent selection bias. Moreover, it is difficult to capture and control all potential confounders. In addition, due to the lack of prior sample size calculation, the sample size may lack power to detect other statistically significant differences in outcomes and prevent the possibility to development a multivariate analysis to evaluate independent predictor, specially LAAO device types, for primary endpoint. Large, well-designed, randomized clinical trials are needed to fully clarify the actual potential benefit of NOAC as antithrombotic treatment after LAAO.

## 5. Conclusions

Post-procedural management of antithrombotic therapy following LAAO remains a challenge. Low-dose apixaban after LAAO may be a good antithrombotic strategy to prevent the incidence of DRT while keeping a favorable safety profile with a low incidence of hemorrhagic events. Randomized trials are warranted.

## Figures and Tables

**Table 1:** Baseline demographics and clinical characteristics.

Characteristic	Total (*n* = 139)	Single Antiplatelet (*n* = 26)	Dual Antiplatelet (*n* = 73)	Apixaban 2.5 mg/12 h (*n* = 40)	*p* Value
Age (years)	73.1 ± 9	75.6 ± 8	71.7 ± 10	74.1 ± 7	0.09
Male gender	89 (65)	18 (69)	47 (65)	24 (60)	0.82
Type of AF					0.82
- Paroxysmal	58 (41)	13 (50)	29 (40)	16 (39)	
- Persistent	3 (2)	0	2 (3)	1 (2)	
- Permanent	78 (57)	13 (50)	42 (57)	23 (59)	
Hypertension	125 (91)	21 (81)	68 (93)	36 (90)	0.16
Previous stroke	49 (36)	11 (42)	18 (25)	20 (50)	0.01
Previous TIA	9 (7)	4 (15)	3 (4)	2 (5)	0.18
Previous major bleed event	106 (77)	23 (89)	53 (73)	30 (72)	0.27
- Gastrointestinal - Intracranial	49 (46) 44 (42)	13 (57) 9 (39)	27 (51) 17 (32)	9 (30) 18 (18)	
- Haematuria	3 (3)	0	2 (4)	1 (3)	
- Epistaxis	5 (5)	1 (4)	2 (4)	2 (7)	
- Respiratory	2 (2)	0	2 (4)	0	
- Others	3 (6)	0	3 (4)	0	
Previous PCI or CABG	34 (25)	7 (27)	20 (27)	7 (18)	0.52
CHA_2_DS_2_-VASc Score	4.3 ± 1.5	4.4 ± 1.4	4.1 ± 1.5	4.5 ± 1.4	0.42
CHA_2_DS_2_-VASc Score ≥ 4	97 (70)	21 (81)	45 (62)	31 (78)	0.06
Baseline Stroke Risk	5.9 ± 2.8	6.1 ± 2.7	5.6 ± 2.9	6.3 ± 2.8	0.42
HAS-BLED Score	3.6 ± 1.0	3.7 ± 0.8	3.5 ± 1	3.7 ± 0.9	0.39
HAS-BLED Score ≥ 3	125 (91)	25 (96)	64 (88)	36 (90)	0.41
Previous AT					<0.001
- None	32 (23)	11 (42)	13 (18)	8 (21)	
- SAPT	45 (33)	8 (31)	32 (44)	5 (13)	
- DAPT	9 (7)	1 (4)	7 (10)	1 (3)	
- AVK	24 (17)	4 (15)	13 (17)	7 (18)	
- NOAC	28 (20)	2 (8)	8 (11)	18 (45)	
- **Low-dose	14 (10)	1 (4)	5 (7)	8 (20)	
Absolute CI to OAC	61 (44)	13 (50)	30 (41)	18 (45)	0.71
- Gastrointestinal	12 (19)	3 (18)	9 (30)	0	0.27
- Intracranial	45 (75)	10 (82)	17 (57)	18 (100)	
- Epistaxis	1 (2)	0	1 (3)	0	
- Respiratory	1 (2)	0	1 (3)	0	
- Others	2 (4)	0	2 (7)	0	
Indication for LAAO					0.11
- Bleeding (prior or high risk)	131 (94)	26 (100)	68 (93)	37 (93)	
- Stroke on VKA	4 (3)	0	2 (3)	2 (5)	
- Patient unwilling VKA	4 (3)	0	3 (4)	1 (2)	

Values are *n* (%) or mean ± SD. AF, atrial fibrillation; TIA, transient ischemic attack; PCI, percutaneous coronary intervention; CABG, coronary artery bypass graft; OAC, oral anticoagulation; AT, antithrombotic treatment; CI, contraindication. **, excluded.

**Table 2:** Procedural characteristics and in-hospital outcomes.

	Total (*n* = 139)	Single Antiplatelet (*n* = 26)	Dual Antiplatelet (*n* = 73)	Apixaban 2.5 mg/12 h (*n* = 40)	*p* Value
Fluoroscopic duration (minutes)	17 ± 9.4	16.5 ± 7	18.1 ± 10	15.1 ± 9	0.27
Contrast (mL)	76 ± 44	79.8 ± 47	80.2 ± 48	65.7 ± 32	0.26
Device type					<0.001
- ACP/Amulet	111 (81)	17 (65)	68 (93)	26 (67)	
- Watchman	3 (2)	2 (8)	1 (1)	0	
- Lambre	24 (17)	7 (27)	4 (6)	13 (33)	
Patients with procedure- or device-related SAEs ≤7 days	4 (4)	0	4 (6)	0	0.16
- Device embolization	0	0	0	0	NA
- Ischemic stroke	0	0	0	0	NA
- Cardiac Tamponade	0	0	0	0	NA
- Vascular access complication	2 (2)	0	1 (1)	0	1.00
- Major bleeding (BARC ≥3)	3 (3)	0	3 (4)	0	0.58
- Death	0	0	0	0	NA

Values are *n* (%) or mean ± SD. SAEs, serious adverse events; ACP, Amplatzer Cardiac Plug; BARC, Bleeding Academic Research Consortium; Major SAEs, death, stroke, embolism, major bleed, device embolization, major vascular complication. Subjects may have had more than one type of major SAE event.

**Table 3:** Clinical outcomes 3-months follow-up.

Clinical Outcome	Total (*n* = 139)	Single Antiplatelet (*n* = 26)	Dual Antiplatelet (*n* = 73)	Apixaban 2.5mg/12 h (*n* = 40)	*p* Value
Efficacy Endpoint (Stroke + SE + DRT)	5 (4)	2 (8)	3 (4)	0	0.25
Safety Endpoint [Major bleeding (BARC ≥ 3)]	7 (5)	0	7 (10)	0	0.03
Composite Endpoint [Efficacy + Safety Endpoints]	11 (8)	2 (8)	9 (12) *	0	0.046
**Secondary Endpoints**					
Ischemic stroke	1 (1)	0	1 (1)	0	1.00
Systemic Embolization	0	0	0	0	NA
Device related thrombus	5 (4)	2 (8)	3 (4)	0	0.25
Any Bleeding (major + minor)	13 (10)	1 (4)	11 (16)	1 (3)	0.06
Mortality	5 (4)	1 (4)	2 (3)	2 (6)	0.84
CV or unknown cause	3 (3)	0	1 (2)	2 (6)	0.20

Values are *n* (%) or mean ± SD. SE = systemic embolization; DRT = device-related thrombus; CV = cardiovascular. * One patient met both endpoints (DRT and a major bleeding).

## Supplementary Materials

The following are available online at https://www.mdpi.com/article/10.3390/jcdd8110142/s1, Figure S1: Antithrombotic treatment after three-months follow-up; Table S1: Clinical outcomes 12-months follow-up.
