# Supplementary material for: Low Dose of Direct Oral Anticoagulants after Left Atrial Appendage Occlusion
Source: J Cardiovasc Dev Dis. 2021 Oct 28;8(11):142. doi: 10.3390/jcdd8110142 (PMC8623304; doi:10.3390/jcdd8110142)

Journal of
Cardiovascular Development and Disease

Table S1. Clinical outcomes 12-months follow-up.

Clinical Outcome	Total (*n* = 132)	No antithrombotic Treatment (*n* = 6)	Single Antiplatelet (*n* = 75)	Dual Antiplatelet (*n* = 39)	Apixaban 2.5mg/12 h (*n* = 12)	*p* Value
Ischemic stroke	1 (1)	0	1 (1)	0	0	0.33
Systemic Embolization	0	0	0	0	0	NA
Device related thrombus	5 (4)	1 (16)	0	2 (5)	0	0.02
Any Bleeding (major + minor)	9 (7)	0	4 (5)	5 (13)	1 (8)	0.53
Major bleeding (BARC ≥ 3)	5 (4)	0	2 (3)	3 (8)	0	0.65
All-cause mortality	6 (4)	0	4 (6)	2 (5)	0	0.73

Values are *n* (%) or mean ± SD.

Figure S1. Antithrombotic treatment after three-months follow-up.

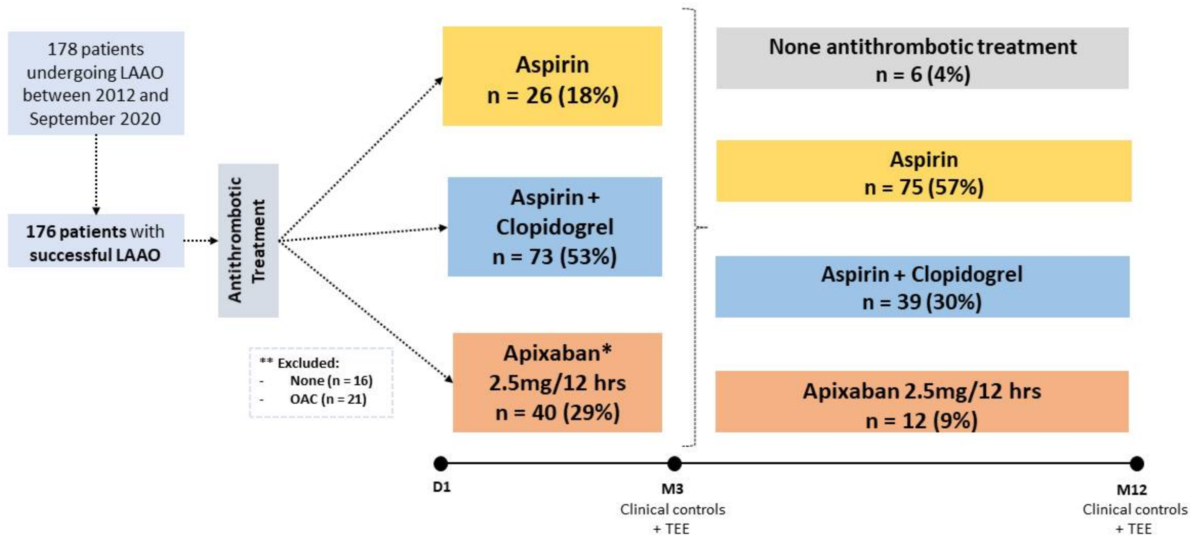

Supplement: Supplementary file 1 [file jcdd-08-00142-s001.zip › jcdd-1380297-supplementary.pdf]
